# Supplementary material for: Cell cycle progression in glioblastoma cells is unaffected by pathophysiological levels of hypoxia
Source: PeerJ. 2016 Mar 3;4:e1755. doi: 10.7717/peerj.1755 (PMC4782743; doi:10.7717/peerj.1755)
Supplement: Table S1 [file peerj-04-1755-s001.pdf]

Table S1. Parameters used for qPCR

Stage	Temperature (°C)	Duration (s)	Ramp rate (°C/s)	Cycles
Pre-incubation	95	600	4.4	1
Amplification	95	5	4.4	45
	60	30	2.2	
Melt curve	95	5	4.4	1
	65	61	2.2	
	97	Continuous	0.11	
Cooling	40	10	1.5	1
