# Supplementary figures and images for: Cell cycle progression in glioblastoma cells is unaffected by pathophysiological levels of hypoxia
Source: PeerJ. 2016 Mar 3;4:e1755. doi: 10.7717/peerj.1755 (PMC4782743; doi:10.7717/peerj.1755)

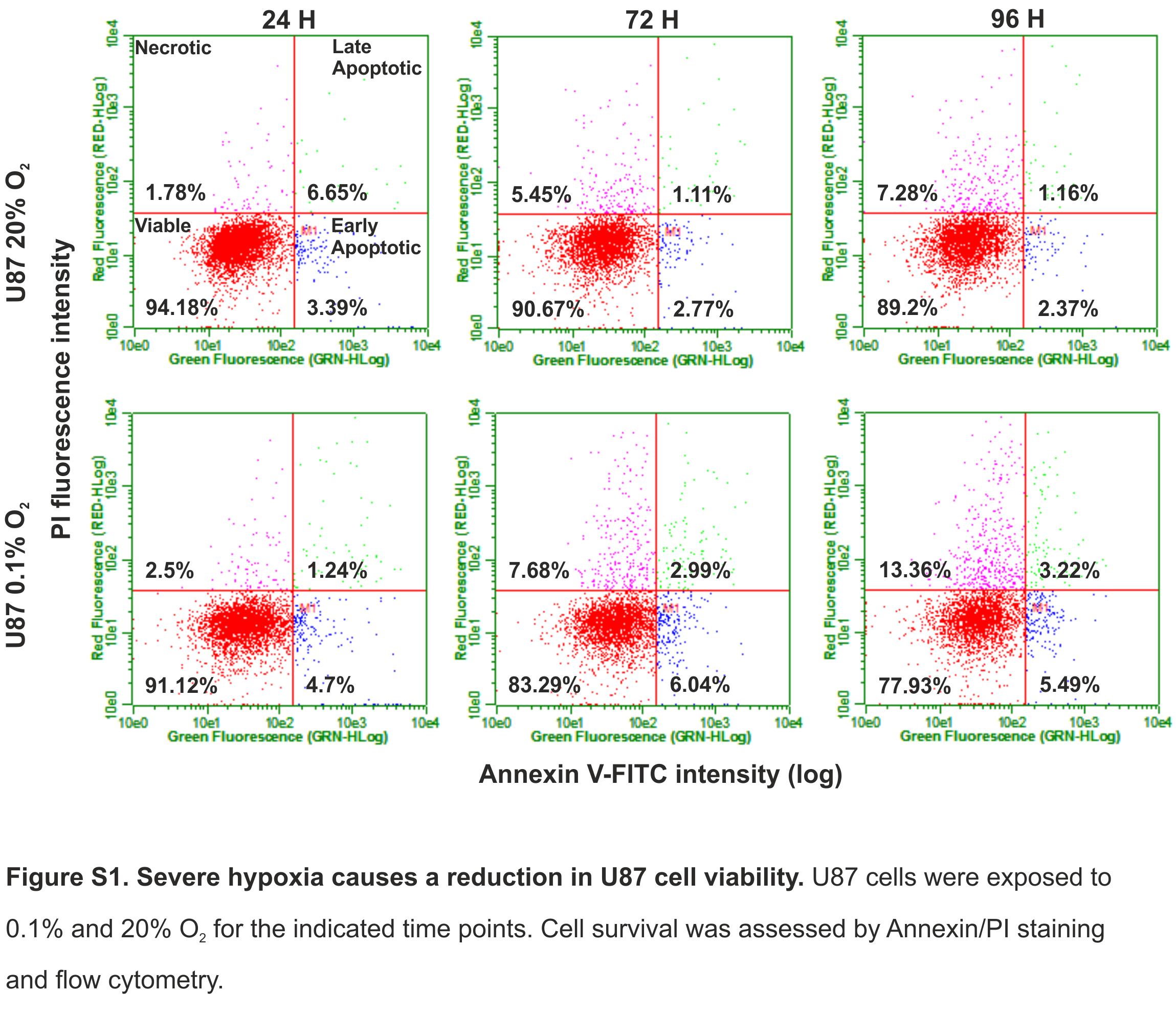

Supplement: Figure S1 — U87 cells were exposed to 0.1% and 20% O2 for the indicated time points. Cell survival was assessed by Annexin/PI staining and flow cytometry. [file peerj-04-1755-s002.png]

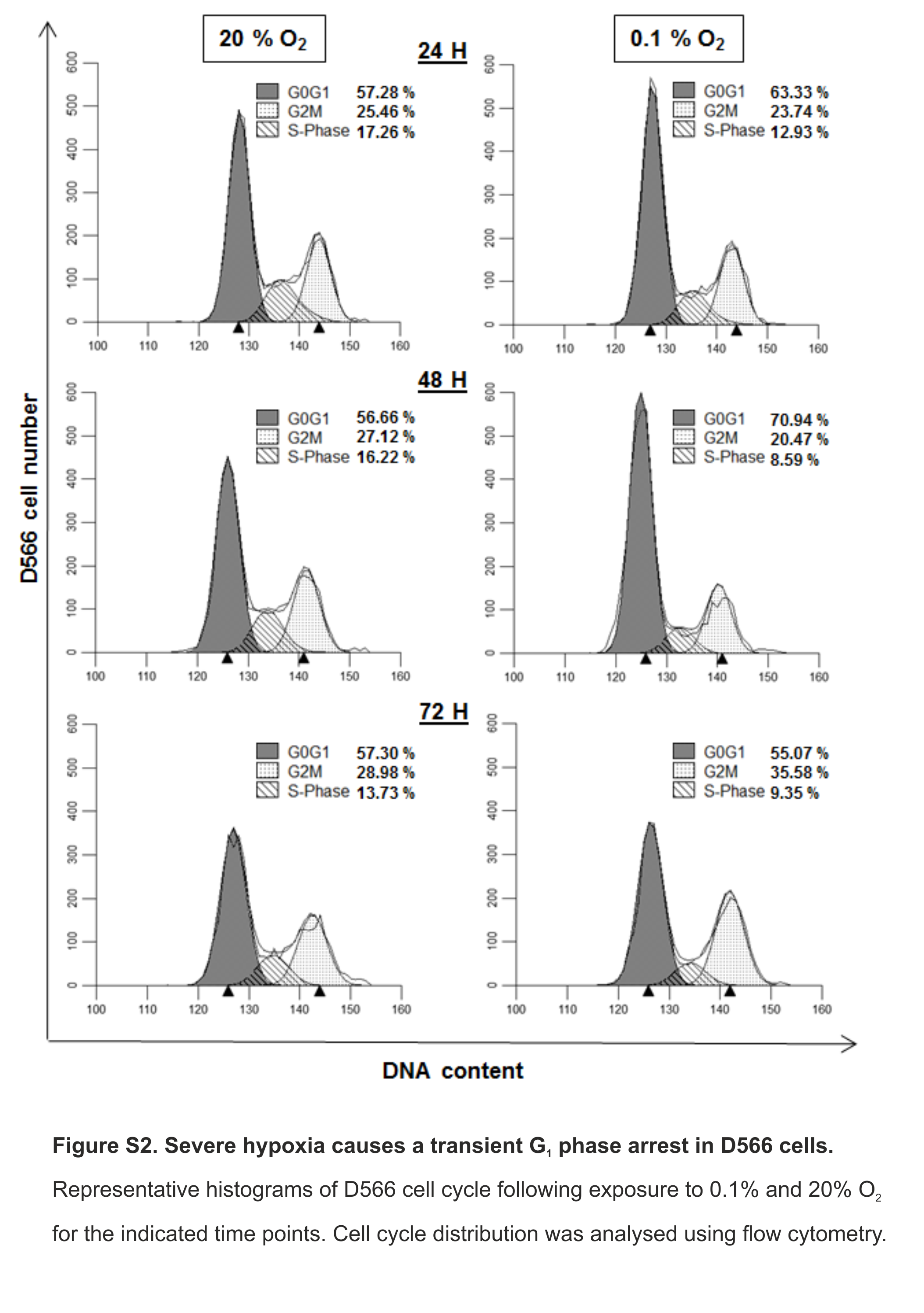

Supplement: Figure S2 — Representative histograms of D566 cell cycle following exposure to 0.1% and 20% O2 for the indicated time points. Cell cycle distribution was analysed using flow cytometry. [file peerj-04-1755-s003.png]

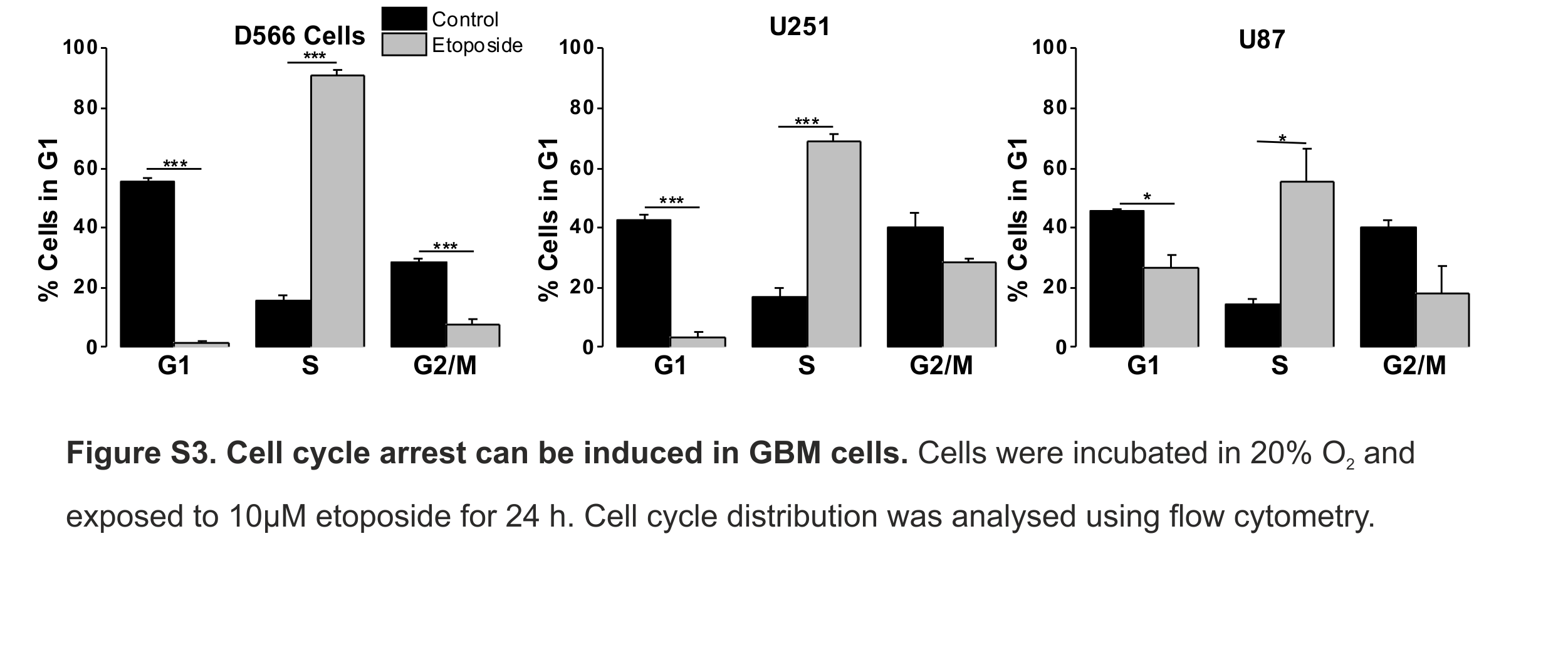

Supplement: Figure S3 — Cells were incubated in 20% O2 and exposed to 10 µM etoposide for 24 h. Cell cycle distribution was analysed using flow cytometry. [file peerj-04-1755-s004.png]
